# Possibility of Using By-Products with High NDF Content to Alter the Fecal Short Chain Fatty Acid Profiles, Bacterial Community, and Digestibility of Lactating Dairy Cows

**DOI:** 10.3390/microorganisms10091731

**Published:** 2022-08-27

**Authors:** Jiaying Lyu, Zhantao Yang, Erdan Wang, Gaokun Liu, Yajing Wang, Wei Wang, Shengli Li

**Affiliations:** State Key Laboratory of Animal Nutrition, Beijing Engineering Technology Research Center of Raw Milk, Quality and Safety Control, College of Animal Science and Technology, China Agricultural University, Beijing 100193, China

**Keywords:** non-forage fiber sources, hindgut, fecal pH, fecal bacteria

## Abstract

This study aimed to investigate whether agricultural by-products with a high NDF content and small-particle-size substitute for forage could cause hindgut acidosis and dysbacteriosis in lactating dairy cows. We investigated the impact of soybean hull and beet pulp on the fecal fermentation, bacterial community, and digestibility of cows. Sixteen lactating Holstein cows were treated as follows (% of dry matter (DM)): amount of by-product added was 0 (control, CON), 1.67% (low by-products, LB), 3.33% (medium by-products, MB), and 5% (high by-products, HB). The results showed the fecal pH of cows to be 7.23–7.29, implying no hindgut acidosis. With increased inclusion of by-products in the diets, the proportion of fecal propionate; relative abundance of the phylum *Bacteroidetes*, the family *Lachnospiraceae*, and genera *unclassified_f_Lachnospiraceae*, *Acetitomaculum*, and *Prevotella*; and the DM and NDF digestibility of cows all increased linearly. Meanwhile, the fecal genera *Turicibacter* and *Clostridium_sensu_stricto_1* decreased linearly. By-products promoted the abundance of fecal bacteria genes related to energy metabolism, glycolysis/gluconeogenesis, and propanoate metabolism; and correlations between fecal short chain fatty acids, digestibility, and the bacteria genera were seen. Overall, our study suggested that adding 5% by-products could be a viable dietary formulation strategy that promotes digestibility and makes positive changes in hindgut fermentation and bacteria.

## 1. Introduction

Agricultural by-products, such as soybean hulls (SHs) and beet pulp (BP), are high in neutral detergent fiber (NDF) and degradability, owing to which they are often used as non-forage fiber sources (NFFSs) in dairy cows’ diets. While NFFSs have similar NDF content as in forage, they have lower lignin and indigestible NDF (iNDF) contents, as well as a smaller particle size. Lactating cows need high-quality roughage to stimulate chewing activities and saliva secretion in order to maintain the rumen pH [1]. Ingesting plenty of forage would increase rumen filling, reduce dry mater intake (DMI), and reduce nutrient digestibility [2], owing to the high content of iNDF in forage. Substituting forage NDF (FNDF) with NFFS-derived NDF can effectively circumvent these challenges, as demonstrated by improved DMI, digestibility, and milk yield in cows fed diets with SH and BP, partially replacing forage [3].

Dairy cows rely on two pathways of the digestive tract to digest feed, namely microbial fermentation in the forestomach and large intestine, and enzymatic processes in the abomasum and small intestine [4]. Generally, hindgut fermentation refers to large-intestine fermentation, where digestion occurs mainly in the cecum. The hindgut microbes of dairy cows are liable for 5% to 10% of carbohydrate degradation [5], which includes starch, small particles that bypass the ruminal fermentation, and components that are undigested in the rumen. The assessment of the fermentation and microbes in feces in dairy cows is a convictive method for the reflection of hindgut microbial ecosystem. Fecal pH, fecal microbial community, and the resulting fermented products, such as short chain fatty acids (SCFA), not only affect the digestion and absorption of nutrients but also change the health status of dairy cows [6]. For example, the hindgut fermentation of plenty of starch that escaped from the rumen might lead to hindgut acidosis [7]. Accumulating evidence has reported epithelial cell damage, endotoxin diffusion, and microbial functional damage in the large intestine when cows have hindgut acidosis [8,9]. Among the factors affecting fecal microbes, such as diet, breed, age, physiological condition, and climate, diet plays the greatest role [6]. As is well-known, the 16S rRNA gene sequence method suggested a high concentrate-to-forage ratio diet to favor proliferation of the phylum *Bacteroides*, while it suggested that a low concentrate-to-forage ratio diet favored the proliferation of *Firmicutes* in the feces of cattle [10].

Studies have consistently shown that replacing corn with BP [11] and adding dried distillers grains [12] in diets could notably change the community structure of fecal microbes in cattle. However, the effects of by-products serve as NFFSs on hindgut microflora, and fermentation in dairy cows has still remained unaddressed. Moreover, the addition of by-products at the expense of forage reduced the particle size and the physical effective NDF (peNDF) content of diet. As a result, the decreased stimulation of salivary secretion by diet in cows could reduce the ruminal pH and induce subacute rumen acidosis (SARA) [13], leading to the weakening of productivity and damage to health. However, by-products replacing forage in diets did not affect ruminal pH [14,15], hence suggesting the dietary formula to possibly not increase SARA risk. However, whether a diet supplemented with SH and BP would allow for more fermentable substrates to be fermented in the hindgut and whether it can induce acidosis and dysbacteriosis in the hindgut are still under investigation.

The current study aimed to evaluate whether replacing forage by SH and BP in diets could affect the fecal SCFA profiles, diversity, and community of fecal bacteria and digestibility of dairy cows. We hypothesized that SH and BP would increase the acidity of the feces and alter the relative abundance of fecal bacteria in cows, depending on the amount of by-products in diets.

## 2. Materials and Methods

### 2.1. Animals and Experimental Design

The experiment was completed in Jinyindao Farm in Beijing (Capital Agribusiness Group, Daxing District, Beijing, China).

A replicated 4 × 4 Latin square design was adopted in this study, with 4 treatments and 4 periods. The four treatment diets were a control group with no by-products and three groups with 1.67%, 3.33%, and 5% by-products replacing 2.2%, 4.4%, and 6.6% forage (alfalfa hay, oat hay, and corn silage), respectively, i.e., CON (control, no by-products), low by-products (1.67%) (LB: 0.83% SH + 0.84% BP), medium by-products (3.33%) (MB: 1.67% SH + 1.66% BP), and high by-products (5%) (HB: 2.5% SH + 2.5% BP) (dry-matter (DM) basis). Each period was 21 days, with 14 days of adaptation and 7 days of data and sample collection. This study used sixteen healthy mid-lactating Holstein dairy cows, which have an average of (mean ± SD) 765 ± 29.33 kg of body weight (BW), 2.44 ± 0.47 of parity, and 186 ± 6.88 d of days in milk (DIM) at the trial initiation. Before the start of the trial, 3 weeks’ milk yield, parity, and DIM of cows were recorded; we divided cows into 4 squares in the order of the three indexes, and each had 4 cows. Cows in each square were assigned to one of the four treatment sequences randomly, and they were raised in the same barn, with free-stall, and had free access to feed and water.

### 2.2. Diets Formulation and Particle Size Distributions

The nutrient levels of the treatment diets was formulated according to the recommendations of NRC (2001) [16] for Holstein cows with 680 kg of BW, 35 kg/d of milk, yield, 4.5% fat, and 3.5% protein. The formulas for the four treatment diets are presented in Supplementary Table S1. The NDF content of four treatment diets was 27%, and the FNDF levels were 18%, 17%, 16%, and 15% of DM in the CON, LB, MB, and HB diets, respectively. A total mixed ration (TMR) machine (Dogo mixer wagon, Storti S.p.A., Italy) was used to cut alfalfa hay, oat hay, and corn silage for 15 min, and a small agitation tank (Runxin Machinery Co., Ltd., Luoyang, China) was then used to mix forage with other concentrate ingredients, water, and wet cane molasses to make TMR.

Throughout the entire trail, the four treatments TMR were sampled weekly. Samples from each time were mixed; for one part, we analyzed the chemical component, and for another, we determined the particle size distributions with the Penn State Particle Separator (PSPS, Nasco, Fort Atkinson, WI, USA). The physically effective factor 8.0 (pef_8.0_) was calculated by dividing the weight of sample kept on the 19.0 and 8.0 mm sieves by total sample weight (% of DM). The physically effective NDF_8.0_ (peNDF_8.0_) was analyzed by multiplying the NDF content with the pef_8.0_ of the diet [13].

### 2.3. Feces Sampling and Fermentation Parameters

On every day during the 17–20 days of each period, we collected fecal samples from each cows’ rectum every 12 h, which reflected fecal samples for every 3 h in 24 h. We immediately measured the pH of the eight samples with a pH meter (Leici Co., Ltd. Shanghai, China), and a part of them was frozen at −20 °C, and another part was frozen at −80 °C. In the lab, the eight fecal samples of each cow in each period stored in −20 °C were thawed and mixed for SCFA analysis; and samples stored in −80 °C were thawed, mixed, and frozen again at −80 °C for bacterial testing.

The SCFA concentration in faces was analyzed by gas chromatograph (Beifentianpu Instrument Co., Ltd., Beijing, China). The fecal samples were thawed and then mixed evenly. A total of 1 g of sample was taken and diluted with 1 mL of water and then centrifuged at 5400 rpm for 20 min, at 4 °C. Then 1 mL of supernatant was mixed with 0.2 mL of 25% metaphosphoric acid solution, which contained 2 g/L internal standard 2-ethylcaproic acid. The mixture was centrifuged at 10,000 rpm for 10 min, at 4 °C, and then the supernatant was collected for measurement. The gas chromatography system was equipped with a 30 m–long fused silica capillary (internal diameter, 0.33 μm; Lanzhou Atech Technologies Co., Ltd., Lanzhou, China).

### 2.4. Chemistry Analyses and Digestibility

The mixed TMR samples and feces samples were dried at 65 °C for 48 h, with an oven (Senxin Instrument Co., Ltd., Huzhou, China). Samples were then ground by using a mill and then passing them through a 1 mm screen (Beijing Kunjieyucheng Machinery Co., Ltd., Beijing, China). The DM contents of the TMR and feces samples, as well as the crude protein (CP), ether extracts, acid detergent lignin (ADL), and ash contents of the TMR samples were determined by using the AOAC, 2005 [17], according to methods 930.0, 942.05, 960.39, 955.04, and 973.18, respectively. The NDF content of the TMR and feces samples and the acid detergent fiber (ADF) contents of the TMR sample were analyzed with a fiber analyzer (Ankom Technology Co., Ltd., Macedon, NY, USA), according to Van Soest et al. [18]. Undigested NDF was used for 240 h of incubation (uNDF_240_) of the contents of TMR and feces samples, which were then measured by an in situ incubations experiment described in the study of Wang et al. [19]; they were used as an indicator for the determination of digestibility of DM and NDF [20] of cows.

### 2.5. DNA Extraction, 16S rRNA Sequence Analysis

The microbial DNA of the feces samples from these dairy cows was extracted with the FastDNA SPIN kit (MP Biomedicals, Solon, OH, USA), in accordance with the manufacturer’s guidelines. The DNA that was extracted was examined with a 1% agarose gel. The amplification of the bacterial 16S rRNA gene region V3–V4 was performed with primer pairs 338F (5′-ACTCCTACGGGAGGCAGCAG-3′) and 806R (5′-GGACTACHVGGGTWTCTAAT-3′) [21], using a PCR thermocycler (ABI GeneAmp^®^ 9700, Foster City, CA, USA). The procedure of PCR amplification for the 16S rRNA gene included the following: 95 °C for 3 min for initial denaturation; 95 °C for 30 s for denaturation with 27 cycles; 55 °C for 30 s for annealing; and 72 °C for 45 s for extension, and then the extension was prolonged for 10 min. The PCR mixture included the following: 5 × TransStart FastPfu buffer, using 4 μL; 2.5 mM dNTPs, using 2 μL; forward and reverse primers, using 5 μM (0.8 μL of each primer); FastPfu DNA polymerase, using 0.4 μL; sample DNA, using 10 ng; and ddH_2_O, using 20 μL. The products of PCR process were extracted by 2% agarose gel; an AxyPrep DNA Gel Extraction Kit (Axygen Biosciences, Union City, CA, USA) was chosen to purify the PCR products in terms of the instructions. The PCR products were quantified with a Quantus™ Fluorometer (Promega, Seattle, WA, USA). The purified amplicons were paired-end sequenced with the Illumina MiSeq platform (Illumina, San Diego, CA, USA) after equimolar pooled. These were based on the specification of Majorbio Bio-Pharm Technology Co., Ltd. (Shanghai, China).

### 2.6. Sequencing Data Processing

The raw reads of 16S rRNA sequencing were demultiplexed with fastp (version 0.20.0) [22] and then combined with FLASH (version 1.2.7) [23]. The 300 bp reads were cut off at the site, receiving an average < 20 of quality over 50 bp sliding window; overlapping sequences longer than 10 bp were aggregated; and then samples were differentiated based on the barcode and primers, and non-exact barcode matched sequences were clustered to an operational taxonomic units (OTUs) by UPARSE (version 7.1) [24]. From each OTU, a single sequence was used as a representative sequence, and its taxonomy was analyzed by using RDP Classifier (version 2.2) [25] against the SILVA v138 16S rRNA database, with a 0.7 confidence threshold. The raw reads of samples were deposited in the NCBI Sequence Read Archive database (Temporary submission ID: SUB11737131, citation accession ID: PRJNA860705).

### 2.7. Statistical Analysis

Phylogenetic investigation of communities by reconstruction of unobserved States (PICRUSt) analyses was used to predict the function of fecal microbes [26] in dairy cows. Data, namely the fecal pH; concentration of total SCFA and SCFA proportion in feces; Alpha diversity indexes; relative abundance of fecal bacterial phylum, family, and genus; relative abundance of the 2 and 3 levels of the Kyoto Encyclopedia of Genes and Genomes (KEGG) pathways; and DM and NDF digestibility of 16 dairy cows in four periods, were tested for normal distribution, using the Proc Univariate of SAS (version 9.4, SAS Institute Inc., Cary, NC, USA), before analysis. The mixed-model procedure in SAS was used for the 4 × 4 Latin square trial design for all of these data in this study, and the model was as follows:

Yijkl = μ + Ti + Pj + Sk + Cl (k) + TPij+ TSik + Eijkl,

where Yijkl is the dependent variable; μ is the overall mean; Ti (i = 1, 2, 3, and 4) is the fixed effect of treatments I; Pj (j = 1, 2, 3, and 4) is the fixed effect of period j; Sk (k = 1, 2, 3, and 4) is the fixed effect of square k; C l(k) (l = 1, 2, 3, ……, 15, and 16) is the random effect of cow l (within square k); TPij is the interaction between treatments i and period j; TSik is the interaction between treatments i and square k; and Eijkl is the residual error. The linear and quadratic effects of increasing by-products in diets were analyzed with the polynomial orthogonal contrasts, using SAS. The mean value of each index was expressed with the least square means, and the differences between treatments were compared by Tukey’s multiple comparison test. Statistical significance of effects was stated at *p* < 0.05, and tendency was stated at 0.05 ≤ *p* ≤ 0.10. The relationships between the fecal fermentation parameters, the DM and NDF digestibility, and the relative abundance of bacterial genera were determined by using Spearman’s correlation test and visualized by the corrplot package [27] of R (version 3.3.0). Only the abundance of bacterial taxa ≥ 0.1% in ruminal samples was analyzed.

## 3. Results

### 3.1. Treatment Diets

The chemical composition of the treatment diets is depicted in Table 1. Substituting forage with by-products at the rate of 1.67, 3.33, and 5% of DM in diets decreased the dietary FNDF content from 18% to 15% of DM. Meanwhile, the contents of dietary ADL, uNDF_240_, and peNDF_8.0_ decreased with the incorporation of by-products into diets. The levels of net energy for lactation (NE_L_), CP, NDF, ADF, and starch in the treatment diets remained consistent.

### 3.2. Fecal Fermentation Profile

The fecal SCFA profile of cows is shown in Table 2. As the amounts of the by-products increased in the diets, the fecal pH; total SCFA concentration; and proportion of acetate, butyrate, and valerate in the feces of the dairy cows did not alter among treatments. In addition, the proportion of propionate increased linearly (*p* = 0.04), while the proportion of isobutyrate decreased linearly (*p* = 0.03) and the proportion of isovalerate tended to decrease linearly (*p* = 0.07) with the increase of by-products in diets of dairy cows.

### 3.3. Alpha Diversity and Bacterial Community in Feces

**Figure 1:**
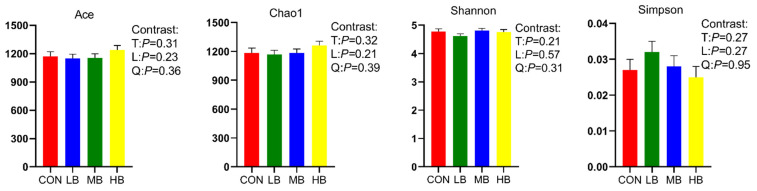
Alpha diversity index of lactating dairy cows fed treatment diets. CON (control, no by-products), low by-products (1.67%) (LB: 0.83% SH + 0.84% BP), medium by-products (3.33%) (MB: 1.67% SH + 1.66% BP), and high by-products (5%) (HB: 2.5% SH + 2.5% BP) (DM basis). T, treatment effect; L, linear effect; Q, quadratic effect.

**Figure 2:**
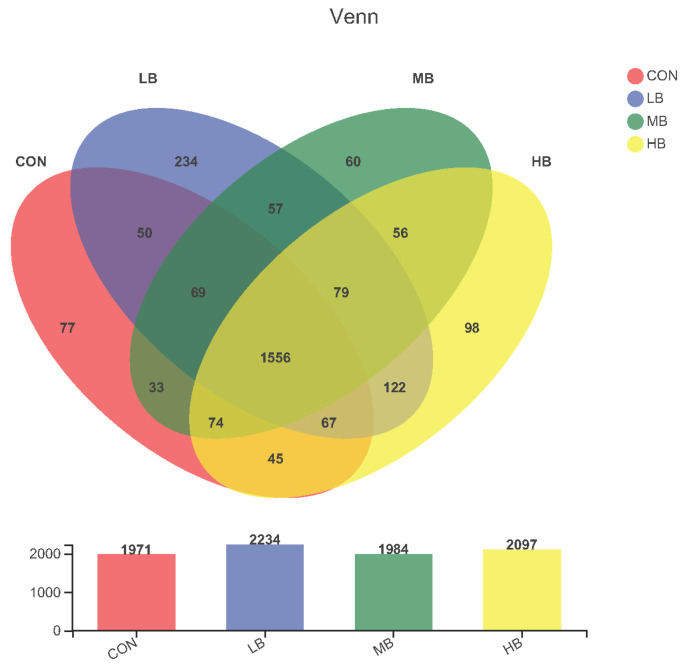
Venn diagram that revealing the relationship of operational taxonomic units (OTUs) of fecal bacteria in cows among treatments. Each circle represents a kind of diet. The number of OTUs shared between the corresponding groups is represented in overlapping areas; the numbers in non-overlapping areas of circles represent OTUs not shared between groups. CON (control, no by-products), low by-products (1.67%) (LB: 0.83% SH + 0.84% BP), medium by-products (3.33%) (MB: 1.67% SH + 1.66% BP), and high by-products (5%) (HB: 2.5% SH + 2.5% BP) (DM basis).

The alpha diversity indexes are shown Figure 1. The alpha diversity remained unaffected by treatment based on the unchanged index ACE, Chao1, Shannon, and Simpson among treatments. A Venn diagram is shown in Figure 2, revealing that each diet showed several unique OTUs, and that 1556 OTUs were shared by the four diets.

In the fecal samples of all cows in four groups, we detected a total of seven bacterial phyla (Table 3); the order of decreasing relative abundance was *Firmicutes* (65.23–71.10%), *Bacteroidetes* (19.23–23.69%), *Actinobacteria* (6.10–7.07%), *Spirochaetes* (1.11–1.77%), *Patescibacteria* (0.47–7.07%), *Proteobacteria* (0.32–0.45%), and *unclassified_ Bacteria* (0.06–0.10%). As the by-products increased in the diets, the relative abundance of fecal *Bacteroidetes* (*p* = 0.04), as well as the *Firmicutes*/*Bacteroidetes* ratio (*p* = 0.03), increased linearly. Moreover, a higher *Firmicutes*/*Bacteroidetes* ratio was found in the feces of cows fed 5% by-products as a replacement for forage than that in cows fed the CON diet (*p* = 0.04). On the contrary, the treatment diets did not alter the relative abundances of *Firmicutes*, *Actinobacteria*, *Spirochaetes*, *Patescibacteria*, *Proteobacteria*, and *unclassified_ Bacteria* in the feces of cows.

A total of 41 families in fecal bacteria were observed across samples; the families whose relative abundance was >0.1% are presented in Table 4. The influence of by-products on the relative abundance of several fecal bacteria families was not significant: the abundance of *Oscillospiraceae*, *Peptostreptococcaceae*, *Rikenellaceae*, *Bifidobacteriaceae*, *Erysipelotrichaceae*, *Christensenellaceae*, *Clostridiaceae*, *Ruminococcaceae*, *Bacteroidaceae*, *Muribaculaceae*, *Eubacterium_coprostanoligenes_group*, and *o__Oscillospirales-f__UCG-010* remained similar between treatments. The family of fecal bacteria was dominated by *Oscillospiraceae* (15.2–15.58%), followed by *Peptostreptococcaceae* (11.06–13.19%) and *Lachnospiraceae* (9.42–12.22%). The relative abundance of the fecal family *Lachnospiraceae* increased linearly (*p* = 0.04), the family *Prevotellaceae* tended to increase linearly (*p* = 0.08), and the family *Bacteroidales_RF16_group* decreased linearly (*p* = 0.02) with the incorporation of dietary by-products increased.

With respect to bacterial genera, the 16s rRNA sequence method revealed the presence of 58 genera in all feces samples of cows, and the relative abundances that are higher than 0.1% are shown in Table 5. The relative abundances of the fecal bacteria genera *Marvinbryantia* (*p* = 0.04), *Acetitomaculum* (*p* = 0.04), and *Prevotella* (*p* = 0.04) were higher in cows fed the HB diet than in those fed the CON diet, the relative abundance of *Dorea (p* < 0.01) was higher in cows fed HB and MB diet than in those fed the CON and LB diets. The relative abundance of *norank_f__Bacteroidales_RF16_group* (*p* = 0.06) tended to be lower in cows fed the HB diet than in cows fed the CON diet. The genera *g__UCG-005*, which belongs to *f__Oscillospiraceae*; *Romboutsia*; *Paeniclostridium*; *Rikenellaceae_RC9_gut_group*, *Bifidobacterium*; *Turicibacter*; and *Christensenellaceae_R-7_group* were the dominant bacteria in feces in dairy cows fed treatment diets. As the amount of by-products increased in the diets, the relative abundance of fecal bacteria genera *unclassified_f__Lachnospiraceae* (*p* = 0.04), *Blautia* (*p* = 0.03), *Acetitomaculum* (*p* = 0.04), *Dorea* (*p* = 0.03), *Prevotella* (*p* = 0.02), and *Prevotellaceae_UCG-001* (*p* = 0.02) increased linearly; and the relative abundance of *Marvinbryantia* (*p* = 0.07), *Lachnospiraceae_NK4A136_group* (*p* = 0.06), and *Prevotellaceae_UCG-003* (*p* = 0.07) tended to increase linearly. The relative abundance of *Ruminococcus* (*p* = 0.01), *unclassified_f__Ruminococcaceae* (*p* = 0.04), *Turicibacter* (*p* = 0.03), *Clostridium_sensu_stricto_1* (*p* = 0.03), and *norank_f__Bacteroidales_RF16_group* (*p* = 0.02) decreased linearly, while the relative abundance of *Cellulosilyticum* (*p* = 0.09) tended to decrease linearly.

### 3.4. PICRUSt Analysis

**Figure 3:**
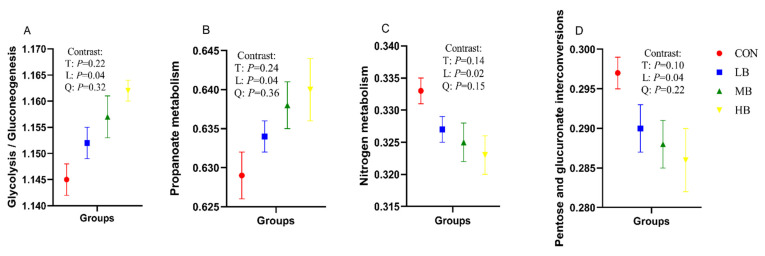
Predicted fecal bacterial function by PICRUSt analysis (KEGG level-3 pathways) of lactating Holstein cows fed diets with by-products substituted for forage. (**A**): Glycolysis/Gluconeogenesis; (**B**): Propanoate metabolism; (**C**): Nitrogen metabolism; (**D**): Pentose and glucuronate interconversions. CON (control, no by-products), low by-products (1.67%) (LB: 0.83% SH + 0.84% BP), medium by-products (3.33%) (MB: 1.67% SH + 1.66% BP), and high by-products (5%) (HB: 2.5% SH + 2.5% BP) (DM basis). T, treatment effect; L, linear effect; Q, quadratic effect.

Table 6 shows the prediction of function of fecal microorganisms in four groups of cows at KEGG level two, using PICRUSt analysis, and the KEGG level-two pathways with relative abundance greater than 1% were analyzed. With the increase of by-products in the diets, the relative abundance of the KEGG level-two pathways for amino acid metabolism (*p* = 0.02), energy metabolism (*p* = 0.03), metabolism of terpenoids and polyketides (*p* = 0.04), and translation (*p* = 0.03) increased linearly; however, the relative abundance of KEGG level-two pathways for metabolism of cofactors and vitamins (*p* = 0.03) decreased linearly. In addition, the relative abundance of KEGG level-three pathways for glycolysis/gluconeogenesis (*p* = 0.04) and propanoate metabolism (*p* = 0.04) with respect to carbohydrate metabolism increased linearly, while pentose and glucuronate interconversions (*p* = 0.04) in carbohydrate metabolism, and nitrogen metabolism (*p* = 0.02) regarding energy metabolism, decreased linearly with the increase of by-products in the diets (Figure 3).

### 3.5. Correlation between Bacterial Genera and Fecal Fermentation Parameter

**Figure 4:**
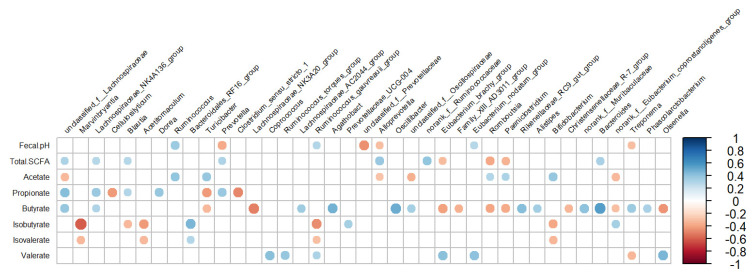
Correlation coefficients between the abundance of bacterial genera (≥0.1%) in feces and fecal pH and short-chain fatty acids for lactating cows fed treatment diets. Blue dots indicate a positive relationship between bacterial genus abundance and production parameters, while red dots represent a negative relationship between them. Larger dots indicate a stronger correlation.

The fecal pH and SCFA profile were correlated with several bacterial genera, which are presented in Figure 4. The fecal pH was positively related to *Ruminococcus* (r = 0.37, *p* < 0.01), while it was negatively related to genera belonging to the family *Prevotellaceae*: *unclassified_f__Prevotellaceae* (r = −0.454, *p* < 0.01) and *Prevotella* (r = −0.376, *p* < 0.01). The total SCFA concentration was positively related to genera belonging to the family *Lachnospiraceae*, such as *unclassified_f__Lachnospiraceae* (r = 0.317, *p* = 0.03), and *Alloprevotella* (r = 0.376, *p* < 0.01), while it was negatively related to *Romboutsia* (r = −0.386, *p* < 0.01). The proportion of acetate was positively related to *Ruminococcus* (r = 0.393, *p* < 0.01) and negatively related to *unclassified_f__Lachnospiraceae* (r = −0.328, *p* = 0.02). Propionate was positively related to genera belonging to the family *Lachnospiraceae*, such as *unclassified_f__Lachnospiraceae* (r = 0.427, *p* < 0.01) and *Dorea* (r = 0.388, *p* < 0.01), while it was negatively related to *Clostridium_sensu_stricto_1* (r = −0.48, *p* < 0.01). Moreover, butyrate was positively related to *Bacteroides* (r = 0.558, *p* < 0.01) and *Oscillibacter* (r = 0.498, *p* < 0.01), while it was negatively related to *Lachnospiraceae_NK3A20_group* (r = −0.5, *p* < 0.01). Isobutyrate and Isovalerate were positively related to *norank_f__Bacteroidales_RF16_group* (r = 0.452, *p* < 0.01; r = 0.293, *p* < 0.01) and negatively related to *Marvinbryantia* (r = −0.6, *p* < 0.01; r = −0.327, *p* < 0.01) and *Acetitomaculum* (r = −0.429, *p* < 0.01; r = −0.326, *p* < 0.01, respectively). Valerate was positively related to *Olsenella* (r = 0.454, *p* < 0.01), but it was negatively related to *unclassified_f__Ruminococcaceae* (r = −0.429, *p* < 0.01).

### 3.6. Digestibility and Correlation between Bacterial Genera and Digestibility

**Figure 5:**
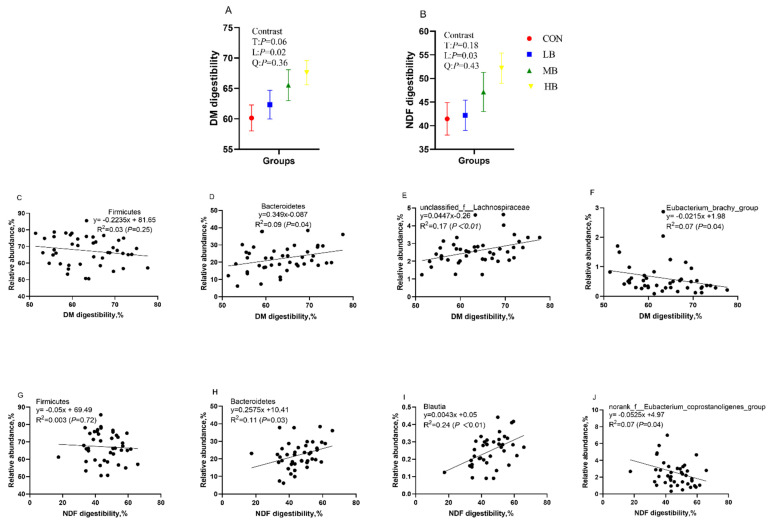
The DM (**A**) and NDF (**B**) digestibility of cows fed treatment diets, the correlation between DM digestibility and the relative abundance of *Firmicutes* (**C**) and *Bacteroidetes* (**D**) at the phylum level, and the relative abundance of *unclassified_f__Lachnospiraceae* (**E**) and *Eubacterium_brachy_group* (**F**) at the genera level; and the correlation between NDF digestibility and the relative abundance of *Firmicutes* (**G**) and *Bacteroidetes* (**H**) at the phylum level and the relative abundance of *Blautia* (**I**) and *norank_f__Eubacterium_coprostanoligenes_group* (**J**) at the genera level. CON (control, no by-products), low by-products (1.67%) (LB: 0.83% SH + 0.84% BP), medium by-products (3.33%) (MB: 1.67% SH + 1.66% BP), and high by-products (5%) (HB: 2.5% SH + 2.5% BP) (DM basis). T, treatment effect; L, linear effect; Q, quadratic effect.

Figure 5 shows the DM and NDF digestibility of dairy cows fed treatment diets and their correlation with the relative abundance of *Bacteroidetes* and *Firmicutes*, as well as with the relative abundance of several bacterial genera. The DM and NDF digestibility increased linearly (*p* = 0.02 and *p* = 0.03; respectively) as the inclusion of dietary by-products increased. The DM and NDF digestibility of cows had no correlation with the phylum *Firmicutes*, but both had a significant positive correlation with *Bacteroidetes* (R^2^ = 0.09, *p* = 0.04; R^2^ = 0.11, *p* = 0.03, respectively). In addition, DM digestibility had a positive relationship with the bacteria genus *unclassified_f__Lachnospiraceae* (R^2^ = 0.17, *p* < 0.01), but it had a negative relationship with *Eubacterium_brachy_group* (R^2^ = 0.07, *p* = 0.04). NDF digestibility had a positive relationship with the bacteria genus *Blautia* (R^2^ = 0.24, *p* < 0.01), but it had negative relationship with *norank_f__Eubacterium_coprostanoligenes_group* (R^2^ = 0.07, *p* = 0.04).

## 4. Discussion

In this study, we evaluated the shifts in fecal fermentation profile, fecal microbial structure, and digestibility of lactating dairy cows ingesting diets containing SH and BP. Compared to forage, SH and BP with low lignin and high degradable NDF (pdNDF) could be rapidly degraded into SCFA in the rumen. In addition, diets supplemented with SH and BP had a small particle size and low content of peNDF, which reduced the stimulation of salivary secretion and the regulation of ruminal pH [13]. Therefore, we hypothesized that replacing forage with NFFS would increase the risk of SARA in dairy cows. However, our companion paper suggested that the duration of pH < 5.6 in the rumen was 9.5–46.7 min/d (unpublished data) [28] in cows fed the four diets, indicating that there was no SARA (ruminal pH < 5.6 persisted for 180 min/d [29]) in the cows in this study. However, the mechanisms of acidosis in the rumen and hindgut are different [7]. Both SH and BP can eventually escape from the rumen and get fermented in the hindgut, increasing the hindgut’s acidosis risk. Since hindgut acidosis is considered to occur when the fecal pH is within a range from 6.0 to 6.6 [30], the 7.23–7.29 range of fecal pH observed in this study suggested no acidosis in the cows. However, we cannot continuously monitor the hindgut pH, owing to the limited experimental condition. Grasping the real-time change of pH is conducive to accurately understanding the environment and health of the hindgut of dairy cows.

The proportion of propionate in the feces increased as by-products’ incorporation into the diets increased; this outcome was in keeping with a previous study that reported that cows fed a diet with reduced particle size have a higher fecal propionate concentration than those fed the control diet [31]. In this study, the NDF level of the treatment diets was the same, although SH and BP had higher ruminal degradability. Therefore, with the increase of by-products in the diet, the dietary NDF flowing into the hindgut decreased, and the non-fibrous carbohydrate (NFC) increased. As a result, bacteria that produce propionate, such as those of the families *Prevotellaceae* [32] and *Lachnospiraceae* [33], grew and proliferated faster, along with the genera *Prevotellaceae_UCG-003*, *Prevotella*, *Prevotellaceae_UCG-001*, *Marvinbryantia*, *Lachnospiraceae_NK4A136_group*, *Blautia*, *Acetitomaculum*, and *Dorea* in feces; these bacteria have the capacity to utilize starch, protein, pectin, and polysaccharides in the diet [34,35]. As a precursor, the increased propionate might contribute to gluconeogenesis in dairy cows. We found a decrease in isobutyrate and isovalerate as increasing by-products in diets; correspondingly, Naderi et al. (2016) had found that replacing forage with 16% (% of DM) BP decreased the proportion of ruminal isovalerate from 1.68% to 0.87% in dairy cows [36]. Isoacids are mainly derived from the decomposition of branched-chain amino acids [37] and are growth factors of cellulose-decomposed bacteria, such as *Ruminococcus albus* [38]. We found that the relative abundance of *Ruminococcus* decreased with the increase of by-products. However, the proportion of isoacids is known to be influenced by both of their production and utilization. Further studies would be required for clarifying the function of isoacids in the digestive tract of dairy cows.

The fecal microbial diversity of cows differs due to a considerable variation in animal age, lactation, genetics, climate, and diet [6]. Changing the composition of the diet may influence the nutrient supply for cows, as well as the available substrates for the microbial environment. Assessing fecal microbes is crucial for investigating the stability of the hindgut microbiota in dairy cows that are fed a diet with SH and BP instead of forages. In our study, the diversity of fecal bacteria of cows was maintained with the increase of by-product inclusion in diets. Castillo-Lopez et al. (2020) reported unaffected fecal bacterial diversity when the particle size of forage in cows’ diet was reduced from 52 to 7 mm [31]. The changes in the bacterial community in the feces suggested a change in fermentable substrate in the hindgut when by-products were added to the diets. Similar to the reports of several researchers [39,40], we observed that *Firmicutes* and *Bacteroidetes* were the most and the second-most predominant phyla in cow feces, accounting for 65.23–71.10% and 19.23–23.69% of the total population, respectively. *Bacteroidetes* are mainly amylolytic bacteria that utilize H_2_, whereas *Firmicutes* are mainly fibrolytic bacteria that produce H_2_ [32]. As the by-products increased, dietary NFCs with a small particle size could possibly escape from the rumen and become fermented in the hindgut, thus accelerating the proliferation of fecal *Bacteroidetes*. Furthermore, the decreased ratio of *Firmicutes*/*Bacteroidetes* with the increase of by-products in the diets indicated the variation of hindgut environment caused by diet to be more suitable for the proliferation of *Bacteroidetes* rather than *Firmicutes*.

At the family level, *Oscillospiraceae* (15.2–15.58%), *Peptostreptococcaceae* (11.06–13.19%), and *Lachnospiraceae* (9.42–12.22%) were the main taxa within the phylum *Firmicutes*, whereas *Rikenellaceae* (6.41–8.49%) and *Prevotellaceae* (4.5–5.41%) were the predominant families of the phylum *Bacteroidetes* in the feces of cows. The dominant families of *Ruminococcacea* (20.19–28.5% [41] or 31.04–32.18% [31]), *Prevotellaceae* (17.8–26% [41]), and *Lachnospiraceae* (11.1–13.7% [41] or 12.17–13.95% [31]) in the feces of lactating Holstein cows have been reported in several studies, and they are inconsistent with the findings of this study. In this study, the family *Oscillospiraceae* had the highest relative abundance in the feces of cows; it mainly produced butyrate [42] and is a kind of beneficial bacteria in the gastrointestinal tract [43]. Moreover, in the current study, the predominant genera were *Oscillospiraceae UCG-005*, *Romboutsia*, *Bifidobacterium*, and *Rikenellaceae RC9 gut group* in the feces of cows. *Turicibacter* is a lactic acid–producing bacterium, with increased relative abundance in the feces of SARA cows [39]. Several reports have shown that *Turicibacter* is a pathogen that can damage animal health [44]. Although *Turicibacter* was reported to be increased in the feces of cows that were fed a small-particle diet [31], the decrease in *Turicibacter* with the increase in dietary by-products that was seen in this study was not expected. Additionally, our results demonstrated that the by-products tended to decrease the percentage of the genus *Clostridium_sensu_stricto_1* in feces, as it has been found to be elevated in the feces of cows subjected to heat stress and was presumed to be a pathogen that may impair intestinal health [45]. The decrease of *Turicibacter* and *Clostridium_sensu_stricto_1* suggested a potential role of by-products in promoting hindgut health in dairy cows. The family *Ruminococcaceae* is a cellulose-digesting taxa [31]; the genera in this family were more abundant in the rumen and in feces of ruminants fed a high-forage diet than in those that were fed a high-concentrate diet [39]. In this study, although NDF contents were identical, in feces, the genera *Ruminococcus* and *unclassified_f__Ruminococcaceae* decreased with the increase of by-products in the diets, perhaps because NDF from SH and BP fermented rapidly in the rumen, and less NDF flowed to the hindgut to affect the abundance of fiber-digesting bacteria.

We evaluated the potential function of fecal bacteria in dairy cows. The most abundant pathways were global and overview maps, carbohydrate metabolism, and amino acid metabolism. These functions are mainly metabolic activities that microorganisms in the hindgut need to perform to survive [46]. The incorporation of by-products in diets promoted the abundance of genes related to energy metabolism, glycolysis/gluconeogenesis, and propanoate metabolism, and this was in line with the change of propionate in feces of the cows. The results indicated that using by-products to replace forage could alter the fermentable substrate in the hindgut and affect the metabolic pathway and fermentation products. However, the accurate function of fecal bacteria needs further study. The correlation between bacterial abundance and SCFA proportion in feces might reflect the bacterial preference for nutrient utilization and the bacterial role in SCFA generation and metabolism. Several genera in the family *Lachnospiraceae* mainly digest dietary NFC to produce propionate [33] and have a positive relationship with propionate. Consistent with the finding in the rumen by Tian et al. [47], the current study indicated that the genus *Prevotella* in the family *Prevotellaceae* was positively correlated with propionate. Additionally, as verified by many previous studies, the production of acetate by *Ruminococcaceae* [31,48] via fiber degradation explained the positive correlation between *Ruminococcus* and acetate in the feces.

The addition of by-products in diets improved the DM and NDF digestibility of cows, an observation that is supported by Miron et al. [3]. BP and SH were degraded more by ruminal microbes, and cows in treatment ingested similar amounts of diets (unpublished data) [28]. As a result, the DM and NDF digestibility of cows were enhanced. The composition and digestibility of the diet have a high impact on the bacterial community in the gastrointestinal tract of ruminants; for example, ruminal *Ruminobacter* and *Oribacterium* were positively and negatively correlated with DM digestibility, respectively [49], and fecal *Fibrobacteres* was positively correlated with the DM digestibility of cows [50]. The DM digestibility of cows was found to be positively correlated with the abundance of the phylum *Bacteroidetes* and genus *unclassified_f__Lachnospiraceae*, whereas they were negatively correlated with the abundance of the genus *Eubacterium_brachy_group*. In addition, NDF digestibility was positively correlated with the abundance of the phylum *Bacteroidetes* and genus *Blautia*, whereas they were negatively correlated with abundance of the genus *norank_f__Eubacterium_coprostanoligenes_group*, in the present study. This correlation suggested that diets with high digestibility favored the colonization of *Bacteroidetes*, and significant abundance of genus *Eubacterium_brachy_group* or *norank_f__Eubacterium_coprostanoligenes_group* in the feces might be detrimental to the digestion of DM or NDF in diets. However, to date, very few research studies have focused on the regulation of digestibility of dairy cows by gastrointestinal bacteria, and further studies are recommended to detect the character of microbes in nutrient digestibility. The limitation of this study was that the epithelial samples of the large intestine could not be obtained to determine the impact of the diets on hindgut health. Diet-induced changes in the hindgut environment, microflora, and intestinal epithelial and their interaction need to be further investigated.

## 5. Conclusions

In this study, we observed that the proportion of propionate, several propionate-producing bacteria in feces, and DM and NDF digestibility of dairy cows increased as the inclusion of by-products increased from 1.67% to 5% of DM. The incorporation of by-products in diets did not cause hindgut acidosis, maintained the fecal bacterial diversity, and positively altered the fecal bacterial community, thereby playing a considerable role in assessing the stability of hindgut microflora and overall health of the cows. Additionally, the function of specific fecal microbes in dairy cows should be considered in future research.

## Figures and Tables

**Table 1:** Chemical composition of treatment diets fed to lactating cows (% of DM, unless otherwise noted).

Item	Treatment Diets ^1^			
	CON	LB	MB	HB
DM, % as fed	52.6 ± 0.27	52.6 ± 0.20	52.6 ± 0.28	52.8 ± 0.20
NE_L_, Mcal/kg ^2^	1.73	1.73	1.73	1.73
CP	16.0 ± 0.27	16.0 ± 0.23	16.1 ± 0.21	16.1 ± 0.29
NDF	27.1 ± 0.23	27.1 ± 0.22	27.1 ± 0.20	27.1 ± 0.20
FNDF ^3^	18.0 ± 0.21	17.0 ± 0.28	16.0 ± 0.30	15.0 ± 0.31
ADF	17.1 ± 0.19	16.9 ± 0.17	16.8 ± 0.20	16.7 ± 0.19
Ether extract	4.45 ± 0.12	4.41 ± 0.10	4.45 ± 0.16	4.48 ± 0.13
NFC ^4^	44.82 ± 0.33	45.09 ± 0.28	45.32 ± 0.32	45.66 ± 0.30
Starch	25.65 ± 0.30	25.72 ± 0.23	25.75 ± 0.33	25.80 ± 0.21
Ash	8.87 ± 0.12	8.79 ± 0.13	8.54 ± 0.18	8.38 ± 0.18
ADL	4.66 ± 0.10	4.44 ± 0.09	4.25 ± 0.12	3.94 ± 0.15
uNDF_240_^5^	9.01 ± 0.11	8.89 ± 0.10	8.51 ± 0.14	8.02 ± 0.11
Separator sieve (% of DM retained on sieve)				
19 mm	11.10 ± 0.34	9.72 ± 0.37	8.43 ± 0.31	7.10 ± 0.33
8–19 mm	34.91 ± 0.28	33.82 ± 0.23	32.52 ± 0.24	31.45 ± 0.27
1.18–8 mm	27.61 ± 0.087	29.91 ± 0.101	32.74 ± 0.092	35.31 ± 0.077
Pan	26.42 ± 0.100	26.54 ± 0.112	26.39 ± 0.073	26.21 ± 0.084
pef_8.0_^6^, %	46.04 ± 0.29	43.63 ± 0.28	40.94 ± 0.23	38.55 ± 0.21
peNDF_8.0_^7^, %	12.52 ± 0.27	11.80 ± 0.27	11.10 ± 0.28	10.48 ± 0.23

Abbreviations: CON (control, no by-products), low by-products (1.67%) (LB: 0.83% SH + 0.84% BP), medium by-products (3.33%) (MB: 1.67% SH + 1.66% BP), and high by-products (5%) (HB: 2.5% SH + 2.5% BP) (DM basis). ^1^ Values were means ± SD, except for NE_L_; n = 6. ^2^ NE_L_= net energy for lactation. NE_L_ was a calculated value according to NRC (2001); ^3^ FNDF = forage NDF. ^4^ NFC was calculated as 100 − [CP% + NDF % + ether extract % + ash %); ^5^ uNDF_240_ = Undigested NDF for 240 h incubation; ^6^ pef_8.0_ = physical effective factor 8.0; and ^7^ peNDF_8.0_ = physical effective NDF 8.0.

**Table 2:** Fecal short-chain fatty acid (SCFA) profile of lactating Holstein cows fed diets with by-products substituted for forage.

Item	Diets ^1^				SEM	*p*-Value		
	CON	LB	MB	HB		Treatment	Linear	Quadratic
Fecal pH	7.29	7.27	7.24	7.23	0.028	0.57	0.16	0.69
Total SCFA, mM	51.09	47.62	53.78	51.79	3.400	0.66	0.62	0.47
VFA proportion, mol/100 mol								
Acetate	73.99	73.89	74.53	73.50	0.589	0.67	0.74	0.40
Propionate	14	14.54	14.58	14.85	0.256	0.18	0.04	0.13
Butyrate	6.52	5.67	5.88	6.06	0.190	0.19	0.21	0.10
Isobutyrate	4.12	3.71	3.47	3.25	0.250	0.06	0.03	0.23
Isovalerate	1.01	0.87	0.79	0.79	0.076	0.17	0.07	0.27
Valerate	0.86	0.86	0.89	0.92	0.054	0.79	0.26	0.97

^1^ CON (control, no by-products), low by-products (1.67%) (LB: 0.83% SH + 0.84% BP), medium by-products (3.33%) (MB: 1.67% SH + 1.66% BP), and high by-products (5%) (HB: 2.5% SH + 2.5% BP) (DM basis).

**Table 3:** The relative abundance of fecal bacterial phylum of lactating Holstein cows fed diets with by-products substituted for forage.

Item	Diets ^1^				SEM	*p*-Value		
	CON	LB	MB	HB		Treatment	Linear	Quadratic
*Firmicutes*	71.10	67.69	65.23	66.11	2.14	0.34	0.13	0.59
*Bacteroidetes*	19.23	20.88	22.99	23.69	1.47	0.20	0.04	0.42
*Actinobacteria*	6.10	7.07	6.53	7.05	1.18	0.92	0.81	0.56
*Spirochaetes*	1.77	1.26	1.54	1.11	0.32	0.55	0.32	0.17
*Patescibacteria*	0.53	0.71	0.47	0.57	0.12	0.42	0.72	0.25
*Proteobacteria*	0.40	0.44	0.32	0.45	0.07	0.49	0.89	0.24
*unclassified_Bacteria*	0.07	0.06	0.08	0.10	0.01	0.22	0.23	0.88
*Firmicutes/Bacteroidetes*	3.92 ^a^	3.23 ^ab^	3.16 ^ab^	2.87 ^b^	0.22	0.04	0.03	0.10

^a,b^ Means within a row among four treatments with different superscripts differ (*p* < 0.05). ^1^ CON (control, no by-products), low by-products (1.67%) (LB: 0.83% SH + 0.84% BP), medium by-products (3.33%) (MB: 1.67% SH + 1.66% BP), and high by-products (5%) (HB: 2.5% SH + 2.5% BP) (DM basis).

**Table 4:** The relative abundance of major fecal bacterial families of lactating Holstein cows fed diets with by-products substituted for forage.

Item	Diets ^1^				SEM	*p*-Value		
	CON	LB	MB	HB		Treatment	Linear	Quadratic
*Oscillospiraceae*	15.58	15.20	15.54	15.36	1.27	0.99	0.96	0.88
*Peptostreptococcaceae*	11.06	13.19	12.26	12.93	1.26	0.49	0.69	0.43
*Lachnospiraceae*	9.42	10.37	10.93	12.22	0.71	0.12	0.04	0.22
*Rikenellaceae*	7.54	6.41	8.49	7.73	0.88	0.27	0.44	0.40
*Bifidobacteriaceae*	5.06	6.10	5.47	5.86	1.16	0.89	0.86	0.60
*Prevotellaceae*	4.50	4.90	5.11	5.41	0.31	0.27	0.08	0.33
*Erysipelotrichaceae*	4.77	6.05	4.61	4.18	0.69	0.30	0.21	0.90
*Christensenellaceae*	4.18	3.54	4.17	3.81	0.35	0.32	0.91	0.28
*Clostridiaceae*	2.92	2.49	2.42	2.71	0.42	0.77	0.67	0.72
*Ruminococcaceae*	2.44	2.58	2.67	2.58	0.24	0.88	0.97	0.76
*Bacteroidaceae*	2.37	2.28	2.15	1.52	0.37	0.43	0.32	0.48
*Muribaculaceae*	2.72	2.36	2.64	2.68	0.30	0.85	0.87	0.67
*Eubacterium_coprostanoligenes_group*	3.12	2.44	2.51	2.12	0.49	0.51	0.31	0.40
*o__Oscillospirales*; *f__UCG-010*	2.62	2.87	2.57	2.32	0.40	0.71	0.77	0.80
*f__Bacteroidales_RF16_group*	1.23	1.03	0.95	0.89	0.088	0.06	0.02	0.22

^1^ CON (control, no by-products), low by-products (1.67%) (LB: 0.83% SH + 0.84% BP), medium by-products (3.33%) (MB: 1.67% SH + 1.66% BP), and high by-products (5%) (HB: 2.5% SH + 2.5% BP) (DM basis).

**Table 5:** The relative abundance of major fecal bacterial genera of lactating Holstein cows fed diets with by-products substituted for forage.

Family	Genus	Diets ^1^				SEM	*p*-Value		
		CON	LB	MB	HB		Treatment	Linear	Quadratic
*Lachnospiraceae*	*unclassified_f__Lachnospiraceae*	2.37	2.50	2.71	2.79	0.110	0.20	0.04	0.45
	*Lachnospiraceae_NK3A20_group*	1.25	1.27	1.31	1.36	0.224	0.53	0.46	0.73
	*Marvinbryantia*	0.42 ^b^	0.45 ^ab^	0.52 ^ab^	0.77 ^a^	0.106	0.04	0.07	0.23
	*Lachnospiraceae_NK4A136_group*	0.50	0.56	0.67	0.69	0.067	0.33	0.06	0.61
	*Coprococcus*	0.41	0.37	0.41	0.46	0.065	0.74	0.67	0.94
	*Ruminococcus_torques_group*	0.57	0.44	0.45	0.46	0.077	0.44	0.41	0.59
	*Lachnospiraceae_AC2044_group*	0.52	0.54	0.50	0.50	0.070	0.97	0.85	0.87
	*Cellulosilyticum*	0.41	0.34	0.32	0.27	0.060	0.41	0.09	0.26
	*Blautia*	0.23	0.22	0.40	0.40	0.056	0.19	0.03	0.98
	*Acetitomaculum*	0.33 ^b^	0.40 ^ab^	0.46 ^ab^	0.52 ^a^	0.053	0.04	0.04	0.37
	*Dorea*	0.29 ^b^	0.25 ^b^	0.42 ^a^	0.46 ^a^	0.055	<0.01	0.03	0.90
	*Ruminococcus_gauvreauii_group*	0.24	0.24	0.28	0.35	0.052	0.12	0.35	0.70
	*Agathobact*	0.17	0.11	0.13	0.15	0.028	0.19	0.90	0.47
	*Frisingicoccus*	0.15	0.21	0.20	0.18	0.029	0.49	0.65	0.55
*Prevotellaceae*	*Prevotellaceae_UCG-003*	1.91	2.27	2.30	2.35	0.131	0.15	0.07	0.20
	*Prevotella*	0.53 ^b^	0.95 ^ab^	0.99 ^ab^	1.11 ^a^	0.132	0.04	0.02	0.11
	*Prevotellaceae_UCG-001*	0.58	0.75	0.78	0.79	0.051	0.11	0.02	0.14
	*Prevotellaceae_UCG-004*	0.399	0.490	0.403	0.298	0.094	0.3	0.94	0.66
	*unclassified_f__Prevotellaceae*	0.364	0.263	0.476	0.334	0.081	0.21	0.87	0.23
	*Alloprevotella*	0.179	0.133	0.127	0.211	0.026	0.11	0.68	0.61
*Oscillospiraceae*	*g__UCG-005*	14.16	13.93	14.25	14.04	1.231	0.98	0.93	0.90
	*g__NK4A214_group*	0.43	0.40	0.41	0.44	0.037	0.81	0.95	0.95
	*Oscillibacter*	0.22	0.24	0.24	0.23	0.032	0.97	0.88	0.83
	*norank_f__Oscillospiraceae*	0.26	0.24	0.27	0.20	0.020	0.15	0.22	0.07
	*unclassified_f__Oscillospiraceae*	0.14	0.14	0.12	0.15	0.015	0.60	0.79	0.34
*Ruminococcaceae*	*Ruminococcus*	1.22	1.17	0.93	0.91	0.088	0.16	0.01	0.70
	*norank_f__Ruminococcaceae*	0.61	0.65	0.77	0.61	0.095	0.47	0.93	0.57
	*unclassified_f__Ruminococcaceae*	0.48	0.44	0.43	0.40	0.023	0.20	0.04	0.23
*Anaerovoracaceae*	*Eubacterium_brachy_group*	0.52	0.46	0.47	0.46	0.060	0.91	0.63	0.79
	*Family_XIII_AD3011_group*	0.45	0.41	0.41	0.41	0.047	0.93	0.72	0.72
	*Eubacterium_nodatum_group*	0.20	0.17	0.16	0.19	0.030	0.69	0.74	0.93
*Peptostreptococcaceae*	*Romboutsia*	6.25	7.68	7.19	7.71	0.771	0.46	0.50	0.33
	*Paeniclostridium*	4.62	5.28	4.77	4.95	0.510	0.66	0.92	0.61
*Rikenellaceae*	*Rikenellaceae_RC9_gut_group*	5.95	4.94	6.75	6.28	0.702	0.23	0.36	0.39
	*Alistipes*	1.43	1.13	1.42	1.37	0.196	0.69	0.75	0.55
*Bifidobacteriaceae*	*Bifidobacterium*	4.86	5.98	5.32	5.71	1.173	0.87	0.85	0.59
*Erysipelotrichaceae*	*Turicibacter*	5.32	4.78	4.48	3.34	0.464	0.18	0.03	0.15
*Christensenellaceae*	*Christensenellaceae_R-7_group*	4.17	3.54	4.16	3.80	0.350	0.32	0.89	0.28
*Clostridiaceae*	*Clostridium_sensu_stricto_1*	3.32	2.63	2.40	2.36	0.251	0.13	0.03	0.21
*Muribaculaceae*	*norank_f__Muribaculaceae*	2.702	2.321	2.629	2.671	0.302	0.83	0.85	0.64
*Bacteroidaceae*	*Bacteroides*	2.37	2.22	2.15	1.68	0.372	0.43	0.32	0.48
*Eubacterium_coprostanoligenes_group*	*norank_f__Eubacterium_coprostanoligenes_group*	1.86	2.26	2.11	2.09	0.362	0.90	0.73	0.56
*Spirochaetaceae*	*Treponema*	1.75	1.24	1.53	1.11	0.313	0.56	0.32	0.17
*Monoglobaceae*	*Monoglobus*	1.15	1.29	1.24	1.25	0.211	0.94	0.74	0.69
*Bacteroidales_RF16_group*	*norank_f__Bacteroidales_RF16_group*	1.23	1.03	0.95	0.89	0.088	0.06	0.02	0.22
*Butyricicoccaceae*	*UCG-009*	0.31	0.28	0.31	0.24	0.053	0.66	0.73	0.51
*Acidaminococcaceae*	*Phascolarctobacterium*	0.22	0.14	0.21	0.22	0.045	0.59	0.72	0.41
*Atopobiaceae*	*Olsenella*	0.10	0.12	0.15	0.15	0.029	0.79	0.29	0.79

^a,b^ Means within a row among four treatments with different superscripts differ (*p* < 0.05).^1^ CON (control, no by-products), low by-products (1.67%) (LB: 0.83% SH + 0.84% BP), medium by-products (3.33%) (MB: 1.67% SH + 1.66% BP), and high by-products (5%) (HB: 2.5% SH + 2.5% BP) (DM basis).

**Table 6:** Predicted fecal bacterial function, by PICRUSt analysis (KEGG level-2 pathways), of lactating Holstein cows fed diets containing by-products instead of forage (relative abundance > 1%).

Item	Diets ^1^				SEM	*p*-Value		
	CON	LB	MB	HB		Treatment	Linear	Quadratic
Global and overview maps	40.40	40.25	40.48	40.44	0.111	0.30	0.43	0.49
Carbohydrate metabolism	9.71	9.68	9.63	9.71	0.042	0.50	0.80	0.64
Amino acid metabolism	7.25	7.27	7.33	7.37	0.032	0.16	0.02	0.39
Energy metabolism	4.15	4.17	4.19	4.20	0.023	0.11	0.03	0.33
Metabolism of cofactors and vitamins	4.32	4.35	4.14	3.89	0.133	0.12	0.03	0.43
Translation	3.86 ^b^	3.92 ^ab^	3.94 ^a^	3.95 ^a^	0.021	0.02	0.03	0.11
Replication and repair	3.37	3.40	3.39	3.40	0.020	0.48	0.38	0.27
Membrane transport	3.04	2.96	2.94	3.02	0.056	0.41	0.52	0.77
Nucleotide metabolism	2.92	2.97	2.94	2.93	0.029	0.32	0.70	0.67
Signal transduction	2.20	2.15	2.18	2.17	0.023	0.16	0.35	0.12
Cellular-community prokaryotes	2.10	2.08	2.08	2.09	0.023	0.66	0.67	0.70
Lipid metabolism	1.86	1.86	1.85	1.85	0.011	0.91	0.41	0.82
Biosynthesis of other secondary metabolites	1.60	1.59	1.61	1.59	0.010	0.39	0.75	0.36
Folding, sorting, and degradation	1.59	1.60	1.59	1.58	0.017	0.69	0.44	0.86
Glycan biosynthesis and metabolism	1.46	1.50	1.50	1.48	0.024	0.31	0.39	0.57
Metabolism of other amino acids	1.22	1.24	1.23	1.24	0.018	0.18	0.31	0.15
Cell motility	1.11	1.05	1.09	1.04	0.037	0.31	0.26	0.13
Metabolism of terpenoids and polyketides	0.99	1.00	1.01	1.02	0.004	0.11	0.04	0.10

^a,b^ Means within a row among four treatments with different superscripts differ (*p* < 0.05). ^1^ CON (control, no by-products), low by-products (1.67%) (LB: 0.83% SH + 0.84% BP), medium by-products (3.33%) (MB: 1.67% SH + 1.66% BP), and high by-products (5%) (HB: 2.5% SH + 2.5% BP) (DM basis).

## Data Availability

The data for this study are not publicly available due to the funder, but they can be obtained by contacting the corresponding author, upon reasonable request.

## Supplementary Materials

The following supporting information can be downloaded at https://www.mdpi.com/article/10.3390/microorganisms10091731/s1. Table S1: Ingredient composition of treatment diets fed to lactating cows (% of DM).
