# Supplementary material for: Possibility of Using By-Products with High NDF Content to Alter the Fecal Short Chain Fatty Acid Profiles, Bacterial Community, and Digestibility of Lactating Dairy Cows
Source: Microorganisms. 2022 Aug 27;10(9):1731. doi: 10.3390/microorganisms10091731 (PMC9505624; doi:10.3390/microorganisms10091731)
Supplement: Supplementary file 1 [file microorganisms-10-01731-s001.zip › microorganisms-1854281-supplementary.pdf]

Supplementary Table

Table S1. Ingredient composition of treatment diets fed to lactating cows (% of DM)

Item	Treatment diets[1]			
	CON	LN	MN	HN
Soybean hull	0	0.83	1.67	2.50
Beet pulp pellet	0	0.84	1.66	2.50
Alfalfa hay	12.3	11.4	10.3	9.2
Oat hay	4.38	3.76	3.00	2.42
Whole corn silage	23.8	23.2	22.8	22.3
Alfalfa silage	2.60	2.60	2.60	2.60
Extruded soybean meal	2.03	2.03	2.07	2.09
Soybean meal	7.11	7.18	7.25	7.32
Steam-flaked corn	27.5	27.7	27.8	28.0
Double-low(low erucic acid, low glucosinolate) rapeseed meal	8.32	8.41	8.48	8.57
DDGS(distillers dried grains with soluble)	0.51	0.53	0.54	0.55
Whole cotton seed	4.17	4.31	4.45	4.58
Wheat bran	1.07	1.09	1.10	1.11
Mineral-vitamin premix[2]	4.02	4.06	4.10	4.14
Fatty acid calcium[3]	0.62	0.60	0.60	0.58
Fatty powder[4]	0.93	0.90	0.90	0.89
Wet cane molasses	0.66	0.67	0.69	0.71

[1]CON (control, no by-products); low by-products (1.67%) (LB: 0.83% SH + 0.84% BP); medium by-products (3.33%) (MB: 1.67% SH + 1.66% BP), and high by-products (5%) (HB: 2.5% SH + 2.5% BP) (DM basis).

[2]The premix for lactating cows contained (per kilogram of DM): 150,000 IU Vitamin A, 35,000 IU Vitamin D, 2,000 IU Vitamin E, 250 mg Cu, 500 mg Mn, 1,000 mg Zn, 20 mg Se, 40 mg I, 25 mg Co.

[3] Fatty acid calcium contained ≥ 82.5% ether extract, ≥ 8% Ca and ≤ 10% ash.

[4] Fatty powder contained ≥ 99.5% ether extract.
